# Defining a “Good Death”: Exploring Veterinarians’ Perceptions of Companion Animal Euthanasia

**DOI:** 10.3390/ani13132117

**Published:** 2023-06-26

**Authors:** Lori R. Kogan, Kathleen A. Cooney

**Affiliations:** 1 College of Veterinary Medicine and Biomedical Sciences, Colorado State University, Fort Collins, CO 80523, USA; kathleen@caetainternational.com; 2 Companion Animal Euthanasia Training Academy, Loveland, CO 80538, USA

**Keywords:** euthanasia, dysthanasia, death, pentobarbital, companion animal, veterinarian

## Abstract

**Simple Summary:**

This study was designed to determine how veterinarians define a good euthanasia experience. Our study included an online survey completed by 249 veterinarians who perform feline and/or canine euthanasia. We found very few veterinarians thought their veterinary school training left them feeling well-prepared to perform euthanasia. When asked to rank a list of factors in terms of perceived euthanasia-related concerns, participants ranked respiratory distress the highest. We found that most veterinarians preferred having owners present during euthanasia and that they want a pain-free, anxiety-free experience for the patient. We have used the results of our study to suggest a new definition of companion animal euthanasia that prioritizes patient welfare along with the needs and expectations of the pet owner.

**Abstract:**

This study was designed to determine how veterinarians define a good euthanasia experience. This information is used to generate a working definition of companion animal euthanasia that aligns with animal welfare standards and pet owners’ expectations. An electronic survey distributed via veterinary-related social media (Facebook, Instagram) and listservs were completed by 249 veterinarians who perform feline and/or canine euthanasia. Our results suggest that very few veterinarians feel their veterinary school training adequately prepared them for euthanasia. When veterinarians were asked to rank a list of physiologic conditions and anatomical traits in order of euthanasia-related concerns, respiratory distress was ranked the highest, while the most concerning physical changes were reported to be indications or impressions of seizures or pain. The most commonly reported euthanasia injection technique performed by participants was intravenous administration of pentobarbital sodium (97%), and most veterinarians preferred having owners present (57%) or having no preference (38%) during euthanasia. Results suggest that veterinarians want a pain-free, anxiety-free experience for the patient, appreciate the use of sedatives before euthanasia, and feel that when available and appropriate, home euthanasia offers several benefits. This understanding of the numerous aspects involved in a good euthanasia experience can help inform the creation of an updated definition of companion animal euthanasia that strives to prioritize the welfare of the patient as well as the needs and expectations of the pet owner.

## 1. Introduction

Companion animal euthanasia is a common procedure in veterinary medicine [1,2,3]. Euthanasia is derived from the Greek terms ‘Eu’, meaning good, and ‘Thanatos’, meaning death. The first recorded use of the word euthanasia is attributed to the Roman historian Suetonius and was used to define a gentle death [4]. The American Veterinary Medical Association (AVMA) guidelines for the euthanasia of animals define euthanasia as “the use of humane techniques to induce the most rapid and painless and distress-free death possible to induce death in a manner that is in accord with an animal’s interest and/or because it is a matter of welfare” [5]. The original meaning of the term euthanasia related more to the experience than to the act of ending life. If positive factors were experienced like loved ones gathered, the dying person was ready and willing, and it was peaceful, a person achieved euthanasia and this was considered a good death [6]. In the late 1800s, the meaning of the word changed to the active form of ending life, a definition we use more commonly today [7]. The word dysthanasia was proposed as a suitable antonym for euthanasia in 2017; however, the term can also mean a lack of adequate medical care before death or prolonging life through medical futility leading to suffering [8].

There are significant and numerous ethical considerations around if and when to euthanize an animal, including conflicts between the interest of the owner and the welfare of the animal, financial constraints, and what constitutes euthanasia vs. ethically justifiable humane killing [9,10,11,12,13,14]. Euthanasia is a procedure that requires technical competency, often in the context of an emotionally charged situation, and performing it correctly is essential to ensure a humane death for animals [15,16]. To offer guidance, several technical guidelines pertaining to appropriate drug protocols and routes of administration have been published [5,17,18]. Yet, euthanasia encompasses both medical and emotional components, including considerations that expand beyond the method itself. There is a growing body of literature that addresses important elements in creating a ‘good death’, including the creation of a low-stress environment and providing clients with adequate information and support [19,20]. Concerns surrounding the animal’s experience during euthanasia, the pet owner’s needs, and the mental health of veterinary professionals all factor in to shape a veterinarian’s approach. In the veterinary context, the term euthanasia is typically used to describe ending the life of an animal in a way that minimizes or eliminates pain and distress [5]. In the context of companion animal medicine, the manner of death has far-reaching implications for pet owners and veterinary personnel [19,21,22]. Veterinarians appreciate the emotional toll it can have on pet owners, as well as themselves [19,23,24]. 

Pet owners’ definition of a good euthanasia experience includes numerous elements that occur well before the procedure begins [25,26]. In a study from 2022, pet owners were asked what differentiates a good from a bad death [25]. The results suggest four procedural components that should be included in companion animal euthanasia: assistance with pre-planning, a pain-free/anxiety-free experience, with the pet in a state of sleep before the final injection is given, and the ability to be present through every step of the procedure [25]. Owners reported wanting assistance with euthanasia pre-planning, such as knowing when the time is right to euthanize, appointment details (e.g., where to gather, who can be present, time of day), procedural elements (e.g., what will happen, what to expect), and aftercare options (e.g., cremation services, burial guidelines). Pet owners also indicated the importance of their pet experiencing little to no pain or anxiety throughout the entirety of the procedure. This is achieved through the use of pre-euthanasia sedation or general anesthesia [18]. The ability to remain together with their pet and be present for every moment of the appointment was also a top priority for most owners. In fact, not being allowed to be with their pet during the entire procedure was viewed as having an extremely (81%) or large (12%) negative effect on their perception of euthanasia [25]. 

Euthanasia has evolved into a pseudo-funeral event where owners are offered the space to openly grieve, gather with loved ones (including other family pets), share stories, and create memorial items [27]. In the field of animal hospice, the veterinary healthcare team considers the needs of the bonded human–animal pair [28]. Euthanasia is the most common means of death for companion animals and the owner’s presence during the appointment continues to rise [3]. Patients appear to have reduced anxiety when kept in contact with their owners and in environments felt to be safe [29,30,31].

Despite the importance of euthanasia, education in companion animal euthanasia in US veterinary colleges is minimal and insufficient [32,33,34,35,36]. A similar perspective has been shared by other countries [15]. This has led many veterinarians to feel their preparation for both the technical and communication aspects of euthanasia is lacking [37]. This study was designed to explore the elements veterinarians feel are important in creating a positive euthanasia procedure for the patient and client. The results, coupled with the findings from our pet owner survey [25], are used to create data-driven recommendations for best practices and a new definition of companion animal euthanasia.

## 2. Materials and Methods

An online, anonymous, cross-sectional survey was designed, reviewed, and tested by the co-investigators and their colleagues. The survey was piloted for ambiguity and/or potentially missing or inappropriate response options with revisions made based on these results. The final survey was approved by the Colorado State University Institutional Review Board (IRB #3379) and distributed through Qualtrics (Qualtrics, Inc., Provo, UT, USA) (see Supplementary Materials). Survey respondents were recruited from 26 April 2022 to 3 October 2022 through veterinary-related social media (Facebook, Instagram) and listservs. 

Veterinarians who indicated they perform euthanasia for dogs or cats were recruited for the study. Demographic information in the form of country, years practicing veterinary medicine, year of graduation from veterinary school, age, gender, race, and ethnicity were collected. Participants were asked to estimate how many euthanasias they have performed (i.e., less than 500, 500–1000, over 1000). They were then asked about their preparation and comfort level with the medical and communication aspects of euthanasia, using a 4-point Likert scale with 1 = not at all/minimal and 4 = very/a great deal. 

Next, respondents were asked to indicate how frequently they use several methods of euthanasia (never/rarely, sometimes, frequently/always) as well as how difficult they feel these methods are for clients to witness (not at all, somewhat, very difficult). They were then asked to rank several physical symptoms and traits in terms of their medical concern when performing euthanasia. They were also asked to indicate their preference for client involvement with euthanasia. 

The next set of questions asked participants to indicate the importance of several factors in creating a good euthanasia experience using a 3-point Likert scale with 1 = not at all/minimally important and 3 = very important. These items were followed by a set of questions pertaining to at-home euthanasia for veterinarians who reported performing euthanasia at home. The survey then asked a series of questions about pre-euthanasia sedatives or anesthetics.

Participants were then asked how several factors related to administering the euthanasia solution would negatively impact their perception of a good death, using a 5-point Likert scale with 1 = no negative impact to 5 = extremely negative impact. Similarly, they were also asked to rate potential patient reactions to euthanasia in terms of negatively impacting the perception of a good death. Using the same 5-point Likert scale, they were then asked to indicate how things that can happen after the death could negatively impact the perception of a good death (e.g., “The patient’s body is not handled with respect after its death.”). 

The next set of questions pertained to elements that may occur during the euthanasia process (e.g., “Client hearing their pet, in what sounds like distress, if out of sight.”). Participants were asked to rate each item based on how it might negatively impact the perception of a good death. Participants were asked if they had experienced a negative euthanasia experience due to medical/technical difficulties, and for those who had, they were asked how they provided support to clients and what (if any) actions they took for future euthanasia.

Data analysis included descriptive statistics to characterize veterinarians’ experiences and views regarding euthanasia. Chi-square was used to assess the association between the year of graduation and comfort level (medical and communication) with euthanasia, how participants felt their veterinary school prepared them for euthanasia, and the importance of euthanasia-related training. Mann–Whitney U nonparametric analyses of variance were used to explore the relationship between the number of years practicing veterinary medicine and how many euthanasia procedures they had performed with several items related to perceptions of euthanasia. Significance level (α) was set at *p* = 0.05 and data were analyzed using SPSS (IBM, Armonk, NY, USA).

## 3. Results

A total of 272 veterinarians responded, of whom 249 reported performing euthanasia for dogs or cats. Because everyone did not answer every question, the total responses for each question have been noted. Most participants were from the United States (182, 73%), female (208, 86%), white (222, 89%), and had been practicing longer than 10 years (181, 73%); 39% (98) had performed over 1000 euthanasia procedures (Table 1).

### 3.1. Veterinary School Preparation

When asked to indicate their comfort level with euthanasia, most participants reported feeling very comfortable with both the medical (183, 74%) and communication (136, 55%) aspects (Table 2). When asked how well they feel their veterinary school/training prepared them for euthanasia, very few reported feeling they were very prepared for either the medical (9, 4%) or communication (8, 3%) aspects. The majority (181, 73%) felt that education about euthanasia techniques and communication are equally important when compared to other clinical skills. When assessing the relationship between the year of graduation and the number of times euthanasia was performed, recent graduates reported performing fewer euthanasia procedures compared to those who had graduated earlier (X^2^ = 80.10 (6), *p* < 0.001). Those who graduated most recently also reported feeling less comfortable with both the medical (X^2^ = 17.52 (6), *p* = 0.008) and communication (X^2^ = 17.00 (9), *p* = 0.049) aspects of euthanasia. No differences based on year of graduation were found for how well participants felt their veterinary school prepared them or the importance of euthanasia-related training. 

### 3.2. Methods of Euthanasia and Perceived Difficulty in Viewing Procedure by Client

Participants were asked to report how often they use a variety of euthanasia methods. Nearly all participants reported using the intravenous method frequently/always (242, 97%) (Table 3). The use of other methods was reported less commonly. When asked to rate the perceived difficulty for clients to witness different methods of injectable euthanasia, participants reported feeling intracardiac the most difficult and intravenous the least difficult to view (Table 3).

When asked about their preference for clients’ involvement in euthanasia, most indicated they preferred clients to be present for the entire procedure (143, 57%), followed by no preference (94, 38%). A small number of participants preferred clients to only be present for the pre-euthanasia sedation (9, 4%), or not present at all (3, 1%). 

**Table 3:** Reported use of euthanasia methods and perception of clients’ difficulty in viewing the method.

	Use				Client Difficulty		
	Never/Rarely	Sometimes	Frequently/ Always		Not at All	Somewhat	Very
Intravenous (n = 249)	1 (1%)	6 (2%)	242 (97%)	Intravenous (n = 248)	126 (51%)	118 (48%)	4 (2%)
Intracardiac (n = 249)	165 (66%)	81 (33%)	3 (1%)	Intracardiac (n = 248)	2 (1%)	42 (17%)	204 (82%)
Intrahepatic (n = 248)	191 (77%)	46 (19%)	11 (4%)	Intrahepatic (n = 247)	35 (14%)	126 (51%)	86 (35%)
Intrarenal (n = 248)	161 (65%)	72 (29%)	15 (6%)	Intrarenal (n = 246)	38 (15%)	126 (51%)	82 (33%)
Intraperitoneal (n = 249)	179 (72%)	67 (27%)	3 (1%)	Intraperitoneal (n = 248)	52 (21%)	144 (58%)	52 (21%)
Oral (n = 246)	241 (98%)	5 (2%)	--	Oral (n = 243)	98 (40%)	84 (35%)	61 (25%)

### 3.3. Euthanasia Drug Injection Administration 

Participants were next asked to rate the importance of several possible effects of euthanasia drug injection on negatively impacting their perception of a ‘good death’. These factors included such examples as “the patient appears scared” and “the veterinary team has to restrain the patient”. The factors rated as having an extremely negative impact by the largest number of participants included “The patient appears to be in pain in their final moments” (199/249, 80%) and “The patient is vocal when the euthanasia solution is injected” (185/249, 74%) (Table 4). When asked how a list of potential reactions during a euthanasia procedure could negatively impact their perception of a ‘good death’, the factors seen as having the largest negative impact included “seizure” (156/249, 63% rated it as an extremely negative impact) and “vocalization” (120/249, 48% rated it as an extremely negative impact).

### 3.4. After Death Factors and Euthanasia Experiences

A short list of factors related to the time immediately after death was given to participants with the instructions to rate how much each factor would negatively impact their perception of a ‘good death’. The factor seen as having the largest negative impact was that the “patient’s body is not handled with respect after death” (rated as extremely negative impact by 177/249, 72%), followed by “the client is not offered adequate time with their pet after death” (122/249, 49%) and “the client is not offered adequate privacy after their pet’s death” (113/249, 45%). 

The next set of questions pertained to possible general euthanasia-related experiences. Examples include “client asked to pay at the front desk after the euthanasia” and “client not allowed to spend time with their pet beforehand”. The factors that are seen as having the largest negative effect on the perception of a ‘good death’ included “client hearing their pet, in what sounds like distress, if out of sight” (rated as extremely negative impact by 191/249, 77%) and “the pet appearing to be in distress when it is returned to the room” (rated as extremely negative impact by 172/249, 69%). The factor seen as having the least negative effect was “client asked to pay at the front desk after the euthanasia” (41/249, 17%) (Table 5). 

### 3.5. Aspects of Euthanasia Experiences That May Increase Likelihood of a ‘Good Death’

A series of euthanasia experience factors were presented to participants with the instructions to indicate how important they felt each factor was in increasing the likelihood of a ‘good death’. Examples of these factors include “pre-planning with the client” and “personnel with advanced euthanasia training”. The factors rated to be ‘a great deal’ in terms of increasing the likelihood of a good death by the most participants included “veterinary team’s communication skills” (242/249, 98%) and “home services” (163/249, 66%) (Table 6). 

### 3.6. Negative Euthanasia Experiences

Most participants (219/249, 88%) reported perceiving at least one negative client experience due to medical or technical difficulties. Those who reported having had a negative euthanasia experience were asked to indicate any and all ways they supported clients afterward. The most common response was a phone call (107/249, 43%), followed by no action taken (56/249, 23%), email (49/249, 20%), and a follow-up visit (20/249, 8%). In regard to a negative euthanasia experience, participants were asked to report what action(s) they took for future euthanasia. The most common response was “altered the technical procedure” (116/249, 47%), followed by “switched to different drugs” (65/249, 26%), “no action taken” (41/249, 17%) and “changed client-present policies” (11/249, 4%). Only a minority of participants reported perceived barriers to making changes in their euthanasia protocols (31/215, 14%). 

## 4. Discussion

Euthanasia is a common means of death for many companion animals where owners are typically offered the opportunity to share their pet’s last moments [27,38]. This study offers several insights into how veterinarians define a good death or positive euthanasia experience. The results of this study can be used to help create a novel definition of euthanasia that highlights the companion aspect of the procedure while maintaining important animal welfare considerations and the needs of those who care for the animal. While pet owners are the most obvious type of companion, anyone who feels a personal bond or emotional connection may be labeled a companion, especially if the nature of the bond leads to emotional hardship during and after euthanasia. As such, addressing companion needs along with the needs of the animal is a vital aspect of a positive euthanasia experience.

Despite the fact that most small animal practitioners will conduct numerous euthanasias over the course of their career, many veterinarians feel inadequately prepared by their veterinary school training. This is likely due to the low number of hours dedicated to the procedure in the curriculum [32,37,39]. While acknowledging that there is limited space within veterinary curricula, nevertheless we feel it important to insert more targeted euthanasia training that includes content related to both technical skills and client communication [37]. Veterinarians’ level of confidence in performing the medical procedure and managing communication components appears to increase during clinical practice years, through direct experience. The more this experience can be provided during schooling, the more prepared new graduates should be and both clients and patients would benefit.

When veterinarians were asked to rank a list of physiologic conditions and anatomical traits in order of euthanasia-related concerns, respiratory distress was ranked the highest. Respiratory distress will lead to patient discomfort and if severe enough, may cause death before the euthanasia solution is administered. Hyperalgesia was also of noted concern due to increased patient sensitivity and the risk of pain from minor handling. Interestingly, edema, low blood pressure, and obesity all ranked lower, despite the fact that each of these factors can make finding veins more difficult for injections. This may be attributed to the participant’s level of skill and comfort in locating veins during euthanasia. In the author’s (KC) experience, veterinary students and recent veterinary graduates find poor venous access a greater concern than more experienced veterinarians. In this study, anatomical traits like shorter veins and excessive skin did not seem to raise concern to any great degree, nor did the brachycephalic (short-nosed) characteristic of some breeds, leading us to believe that the concern of respiratory distress is more specific to disease processes like congestive heart failure or respiratory compromise. When seeking a good death for patients, veterinarians may elect to be more proactive to avoid significant respiratory distress in patients (e.g., offer oxygen support, perform euthanasia sooner, and use opioids to reduce dyspnea).

Most veterinarians in our sample preferred having owners present during euthanasia, perhaps because they feel it improves patient comfort (reduction of anxiety) and offers the opportunity to better understand the relationship between owner and patient [29,40,41]. A survey of pet owners [25] found that being present, or at least being allowed to be present (even if they declined the option), was very important. Best practices to help maintain a strong owner-pet connection during euthanasia include offering the option for owners to be present, as well as improved technique delivery, enhanced communication skills, and elevated staff emotional intelligence. If there is concern about whether the owner wishes to remain present for the euthanasia, a brief discussion regarding what they want and what to expect during the procedure may be helpful during a pre-euthanasia visit. Both pet owners and veterinarians acknowledge that the dying pet will exhibit physical changes, and that some may be more undesirable than others. The most concerning physical changes for both owners and veterinarians are any indications or impressions of seizures or pain. If a seizure occurs before euthanasia is performed, it may be advisable to wait for it to conclude before proceeding, to avoid the seizure as the owner’s last memory of their pet. Veterinarians will need to address this on a case-by-case basis. Active signs of dying like body stretching, muscles twitching, and tongue protruding are seen as less concerning to veterinarians compared to owners. This is likely due to veterinarians’ familiarity with active signs of death and the knowledge that these changes are normal and harmless. To the untrained eye, however, any physical change during death may be concerning and should be explained, albeit gently, with simple language to avoid the impression of a dysthanasia [28].

Participants’ responses in the current study aligned well with pet owners’ responses [25] on factors related to home euthanasia. Pet owners ranked home euthanasia experiences as preferable over other locations. Both groups reported wanting to start on time and the need to make euthanasia a personal and private experience. Pet owners seek compassion and confidence from their veterinarians, and the intent to keep their pets comfortable and pain-free [25]. This sentiment includes the use of pre-euthanasia sedatives or anesthetics, both very common in all euthanasia settings. Veterinarians are best served by having the necessary supplies and materials ready to successfully complete the procedure in a pain-free manner in a location within the home deemed most ideal by the pet owner. 

In addition to offering home euthanasia services, veterinarians feel that strong communication skills can lead to better euthanasia experiences for pet owners and patients. Overall, participants did not report feeling that the provision of pre-visit pharmaceuticals (sedatives) makes a large positive impact; however, pet owners want options to reduce their pet’s stress during the experience and oral sedatives can play an appropriate role [42].

The most common euthanasia injection technique used by participants of the study was the intravenous administration of pentobarbital sodium. The method participants report having the most concerns about performing in front of owners was intracardiac injection. It is not known, however, why the presence of pet owners during intracardiac injections is viewed less favorably. It might be due to patients not being adequately anesthetized or the possible presence of visible blood in the syringe during the injection, although modern techniques keep blood hidden from view [17]. It might also be due to the perception that the injection is deeper and more invasive than in a peripheral vein and/or the need to repeat needle insertion multiple times before the drug can be injected. Pet owners have shared that as long as their pet is pain-free and peaceful, they are receptive to veterinarians choosing whichever technique they prefer [25] and veterinarians agree that changing a euthanasia method during the appointment is an acceptable and often necessary protocol.

Most veterinarians in our study indicated the amount of time that transpires between pre-euthanasia drug administration and the euthanasia injection is between 3 and 10 min, the same amount of time expected by pet owners, although owners reported that taking longer is acceptable as long as the pet was comfortable [25]. In other words, a fast death does not necessarily equal a good death. To further explore how veterinarians define a good death for pet patients, we asked them to rank in importance several related factors. Appropriate patient response to drug injections ranked highest and any added cost to euthanasia based on medical decisions made in the best interest of the patient ranked lowest. This suggests most veterinarians feel that while some medical aspects of euthanasia may cost more, as long as they are of benefit to the animal, they are felt to be worth it. Examples include interventions aimed at preventing some of the more alarming effects of pre-euthanasia drugs or euthanasia solutions (e.g., seizures, vocalization, painful injections). 

Other elements that are seen as contributing to a negative euthanasia experience from the veterinarians’ perspective include if pet owners hear their pet in distress from another room (e.g., when an indwelling intravenous catheter is being placed in the leg for the procedure), or the pet appears in distress when it is returned to the euthanasia room. These elements can be eliminated by keeping the patient with the owner for the duration of the appointment and providing intramuscular or subcutaneous pre-euthanasia sedatives before further restraint is needed. Although not seen as detrimental as seeing their pet in distress, many veterinarians reported feeling that requiring pet owners to pay upfront for services following the appointment can negatively impact the experience. It is recommended that owners pay for euthanasia, when possible, before or at the start of the appointment [43,44,45]. Clients asked to pay money when they have just euthanized their pet may tip the scales from being a positive experience to a negative one.

Following a technically difficult euthanasia appointment (e.g., pain, trouble administering drugs), over 75% of veterinarians reported consoling pet owners. Contacting pet owners to review what happened can relieve owners’ concerns regarding their pet’s mental and physical state immediately preceding death [46]. The majority (83%) of veterinarians who experienced a technically difficult euthanasia reported taking some type of action to alter the procedure (e.g., adjusting the drugs and/or technique for future patients). Only 4% of veterinarians indicated they changed their policy on who could and could not be present during euthanasia. Dissuading or preventing owners from being present for the euthanasia of their pet is not advised, and conflicts with what most pet owners prefer [25,26]. It is suggested that euthanasia clinical practice guidelines should be designed to include policies on how to manage difficult euthanasias, how to speak with pet owners about what occurred, and how to prevent similar episodes in the future. 

Results of this study suggest that veterinarians share similar views with pet owners pertaining to what defines a good death experience. Veterinarians listed good communication as an important factor for a quality euthanasia experience and pet owners expressed the desire for pre-planning. Veterinarians are therefore encouraged to communicate openly and early in the decision-making process to assist owners in preparing for euthanasia, including after-death body care wishes [47]. Veterinarians can meet pet owner desire for respectful body handling by avoiding the use of trash bags to hold deceased pets and expediting aftercare with adherence to owner requests [47]. Owners should also be offered time alone with their pet both before and after the procedure. One way to increase such opportunities is to expand the time allotted for euthanasia appointments. Extra time becomes available for privacy and reduces one’s tendency to rush through the sensitive elements of the procedure.

Veterinarians and pet owners want a pain-free, anxiety-free experience for the patient. Both appreciate the use of sedatives before euthanasia, although based on this study, the use of pre-visit sedative pharmaceuticals can be increased to further improve patient comfort. Both understand the benefits of home euthanasia and while there are times it is not an option, pet owners and veterinarians would be well served to discuss this option well in advance of the procedure. Leveraging home euthanasia practitioners within the community can help increase the likelihood of a home visit when the time comes. Finally, most veterinarians indicated they prefer when pet owners are present for euthanasia. Pet owners want the option to be in the room and expressed a strong desire to not be separated from their pet at any time during the euthanasia appointment. The following recommendations are made based on the results of this study and the aforementioned pet owner study [25].

Veterinary Team Recommendations to Improve Companion Animal Euthanasia

1. Provide time to discuss owner options, assist with pre-planning, and collect payment in advance when appropriate;
2. Expand appointment time to allow for all the necessary elements, including owner grieving time;
3. Leverage home euthanasia where possible and have supplies readily available;
4. Focus on patient comfort before and during the procedure;
5. Place added effort on avoiding pain and anxiety;
6. Provide pre-euthanasia sedation or anesthesia before restraint;
7. Keep owner and patient together; avoid separation;
8. Describe the procedure and signs of death in simple language;
9. Utilize whichever methods are most appropriate for the patient and situation, including intracardiac injections;
10. Take time to get the details right and remain compassionate;
11. Handle the deceased body with respect.

There are several limitations to this study. This was a small sample size, obtained online, so veterinarians who feel strongly about the topic may have been more likely to respond to the survey. While online surveys are suitable for a cross-sectional collection of information [48], biases due to self-selection [49], where the study is advertised [50], under-coverage, non-response, and sampling error force caution when interpreting the results [51,52]. 

Additionally, one of the listservs used to collect participants focuses on euthanasia education (KC is affiliated with an organization that offers euthanasia training and has written a book on home euthanasia), so the percentage of respondents who perform at-home euthanasia may not be representative of the general veterinary population. For example, in our sample, 59% reported performing in-home euthanasia, but this number might be artificially enlarged due to our sample. It is also possible that the online format led to a greater likelihood of younger veterinarians completing the survey. All these limitations suggest caution when generalizing to other veterinarians. In general, while this study did find many parallels between pet owners and veterinarian definitions of a ‘good death’, there remain opportunities to explore the definition of dysthanasia. 

## 5. Conclusions

Using the information gleaned from this study and our previous research pertaining to pet owners [25], we feel it is possible to craft a clear definition of companion animal euthanasia that meets the expectations of both veterinarians and pet owners and provides the gentle death each patient deserves. Our goal was to expand on the current definition of euthanasia to incorporate the needs of the companion as well as the animal patient. With these elements in mind, we suggest the new definition of companion animal euthanasia to be ‘The proper use of techniques to end the life of an animal with minimal to no pain or anxiety, best facilitated through use of sedatives/anesthetics, that allows the informed pet owner to remain present for the entirety of the procedure.’ Dysthanasia or bad death experience will be the opposite of this definition, comprising: ‘The inability for owners to be present, a lack of pre-euthanasia sedation, and any pain or distress of the patient observed during the euthanasia procedure.’ Dysthanasia may be prevented by focusing on what leads to the safest and most comfortable experience for both patient and owner. These definitions can be used to shape educational curricula, hospital training, and continuing education for all veterinary personnel including licensed veterinary technicians, with the desired result of increasing the likelihood of a peaceful and incident-free euthanasia experience for all involved. 

## Figures and Tables

**Table 1:** Participant demographics.

	United States	Canada	United Kingdom	Australia	Other		
Country (n = 249)	182 (73%)	46 (19%)	4 (2%)	3 (1%)	14 (6%)		
	Female	Male	Non-binary	NA			
Gender (n = 242)	208 (86%)	27 (11%)	1 (0.5%)	6 (3%)			
	Asian	Black/ African American	Biracial/ Multiracial	Native American/ Alaskan Native	Native Hawaiian/ Pacific Islander	White	NA
Race (n = 248)	4 (2%)	0	4 (2%)	2 (1%)	1 (0.5%)	222 (89%)	15 (6%)
	Hispanic or Latino	Not Hispanic or Latino	NA				
Ethnicity (n = 224)	17 (8%)	190 (85%)	17 (8%)				
	Under 30 years of age	30–39 years old	40–49 years old	50–59 years old	60 years or older	NA	
Age (n = 243)	19 (8%)	62 (26%)	68 (28%)	58 (24%)	35 (14%)	1 (0.5%)	
	Less than 5 years	5–10 years	Longer than 10 years				
Years practicing veterinary medicine (n = 249)	40 (16%)	28 (11%)	181 (73%)				
	1970–1989	1990–1999	2000–2009	2010–2022			
Year graduated veterinary school (n = 249)	37 (15%)	51 (21%)	71 (29%)	90 (36%)			
	Less than 500	500–1000	More than 1000				
Number of euthanasia performed (n = 249)	79 (32%)	72 (29%)	98 (39%)				

**Table 2:** Reported comfort level and perception of veterinary training of medical and communication aspects of euthanasia (n = 249).

	Not at All/ Minimal	Some	Quite a Bit	Very/ a Great Deal
Comfort level with medical aspects of performing euthanasia	0 (0%)	4 (2%)	62 (25%)	183 (74%)
Veterinary training/school preparation for medical aspects of euthanasia	84 (34%)	109 (44%)	47 (19%)	9 (4%)
Comfort level with communication aspects of performing euthanasia	4 (2%)	28 (11%)	81 (33%)	136 (55%)
Veterinary training/school preparation for communication aspects of euthanasia	130 (52%)	86 (35%)	25 (10%)	8 (3%)
	Less important	Equally important	More important	
Importance of euthanasia techniques and euthanasia communication training compared with other clinical skills	22 (9%)	181 (73%)	46 (19%)	

**Table 4:** Rated importance of potential aspects of euthanasia drug injections in creating a negative effect on the perception of an overall ‘good death’ experience (n = 249).

	No Negative Impact	Small Negative Impact	Moderate Negative Impact	Large Negative Impact	Extreme Negative Impact
Patient appears to be in pain in final moments	0 (0%)	4 (2%)	5 (2%)	41 (17%)	199 (80%)
Patient is vocal when euthanasia solution injected	1 (<1%)	0 (0%)	16 (6%)	47 (19%)	185 (74%)
Patient thrashes or makes sudden movements when euthanasia solution injected	1 (<1%)	0 (0%)	15 (6%)	56 (23%)	177 (71%)
Patient experiences seizure	1 (<1%)	7 (3%)	22 (9%)	63 (25%)	156 (63%)
Patient exhibits vocalization at anytime during through procedure	3 (1%)	8 (3%)	36 (15%)	82 (33%)	120 (48%)
Client does not have opportunity to be physically close to pet during last minutes	2 (1%)	10 (4%)	37 (15%)	90 (36%)	110 (44%)
Patient appears scared	2 (1%)	14 (6%)	47 (19%)	86 (35%)	100 (40%)
Patient seems stressed	1 (<1%)	12 (5%)	41 (17%)	102 (41%)	93 (37%)
Veterinary team has to restrain patient	3 (1%)	28 (11%)	55 (22%)	85 (34%)	78 (31%)
Veterinary team has to give more euthanasia solution after the first injection or resort to another method	4 (2%)	51 (21%)	88 (35%)	59 (24%)	47 (19%)
Veterinary team cannot easily find a vein	5 (2%)	34 (14%)	101 (41%)	66 (27%)	43 (17%)
Patient exhibits agonal breathing	16 (6%)	72 (29%)	78 (31%)	50 (20%)	33 (13%)
Patient’s death not as fast as expected	9 (4%)	54 (22%)	88 (36%)	70 (28%)	27 (11%)
Patient’s death appears abrupt	31 (12%)	74 (30%)	67 (27%)	54 (22%)	23 (9%)
Patient regurgitates/vomits	5 (2%)	55 (22%)	92 (37%)	81 (33%)	16 (6%)
Veterinary team has to deviate from plan but reason explained to client	19 (8%)	102 (41%)	93 (37%)	28 (11%)	7 (3%)
Patient exhibits body stretching	27 (11%)	99 (40%)	85 (34%)	28 (11%)	10 (4%)
Patient exhibits muscle twitches	43 (17%)	139 (56%)	48 (19%)	16 (6%)	3 (1%)
Patients’ tongue protruding out of the mouth	81 (33%)	127 (51%)	31 (12%)	7 (3%)	3 (1%)
Patient’s eyes stay open	99 (40%)	104 (42%)	38 (15%)	7 (3%)	1 (<1%)
Patient urinates/defecates	72 (29%)	118 (47%)	48 (19%)	11 (4%)	0 (0%)

**Table 5:** Rated importance of possible aspects of euthanasia in creating a negative effect on the perception of an overall ‘good death’ experience (n = 249).

	No Negative Impact	Small Negative Impact	Moderate Negative Impact	Large Negative Impact	Extreme Negative Impact
Client hearing their pet, in what sounds like distress, if out of sight	0 (0%)	0 (0%)	8 (3%)	50 (20%)	191 (77%)
The pet appearing to be in distress when it is returned to the room	0 (0%)	0 (0%)	11 (4%)	65 (26%)	172 (69%)
Client not allowed to be with their pet during the entire procedure	2 (1%)	9 (4%)	40 (16%)	54 (22%)	144 (58%)
Client not allowed to spend time with their pet beforehand	0 (0%)	7 (3%)	29 (12%)	82 (33%)	131 (53%)
The process is not explained appropriately to client’s children	2 (1%)	18 (7%)	39 (16%)	86 (35%)	103 (42%)
Client feeling that the appointment is too short	2 (1%)	14 (6%)	53 (21%)	102 (41%)	78 (31%)
Client not allowed to have children present	8 (3%)	28 (11%)	60 (24%)	80 (32%)	73 (29%)
Client not allowed to have other pet(s) present	12 (5%)	40 (16%)	80 (32%)	65 (26%)	50 (20%)
Client not allowed to bring their own music, candles, or other items	8 (3%)	49 (20%)	70 (28%)	80 (32%)	42 (17%)
Client asked to pay at the front desk after the euthanasia	25 (10%)	43 (17%)	66 (27%)	74 (30%)	41 (17%)

**Table 6:** Rated aspects of euthanasia experiences in terms of increasing the likelihood of a ‘good death’ (n = 249).

	None at All/Minimal	Somewhat	A Great Deal
Veterinary team’s communication skills	0 (0%)	6 (2%)	242 (98%)
Home services	3 (1%)	81 (33%)	163 (66%)
Pre-planning with the client	9 (4%)	76 (31%)	163 (66%)
Personnel with advanced euthanasia training	19 (8%)	75 (30%)	154 (62%)
Euthanasia attendants (consistent person with client for duration)	33 (13%)	106 (43%)	108 (44%)
Patient pre-visit pharmaceuticals	31 (13%)	125 (50%)	92 (37%)

## Data Availability

Data available on request.

## Supplementary Materials

The following supporting information can be downloaded at: https://www.mdpi.com/article/10.3390/ani13132117/s1, Canine and Feline Euthanasia Survey-Veterinarian.
