# Supplementary material for: Defining a “Good Death”: Exploring Veterinarians’ Perceptions of Companion Animal Euthanasia
Source: Animals (Basel). 2023 Jun 26;13(13):2117. doi: 10.3390/ani13132117 (PMC10339858; doi:10.3390/ani13132117)
Supplement: Supplementary file 1 [file animals-13-02117-s001.zip › animals-2408208 - Supplementary file.pdf]

# Canine and Feline Euthanasia Survey-Veterinarian

Euthanasia experiences

We are interested in how you, as a veterinarian, define a “good” and “bad” euthanasia experience. This survey applies to Pentobarbital Sodium euthanasia administration only and is specific to canine and feline euthanasia.

Euthanasia is derived from the Greek terms ‘eu’ meaning good and ‘thanatos’ meaning death. The term euthanasia is typically used to describe ending the life of an individual animal in a way that minimizes or eliminates pain and distress; an important aspect of animal welfare. In the context of companion animal medicine, the manner of death has far-reaching implications for those present including clients and veterinary personnel.

This study explores what veterinarians feel is important in creating a safe and meaningful euthanasia procedure for the patient, client, and veterinary team beyond the medical act of euthanasia itself. We plan to use results from this study to build data driven recommendations for best euthanasia practices.

If you are a veterinarian who euthanizes dogs and/or cats, we would like to ask you to take the following anonymous survey. Participation will take approximately 10-15 minutes.

Additional study details:
My name is Lori Kogan and I am a researcher from Colorado State University in the Clinical Sciences department and Principal Investigator of this study. We are conducting a research study to learn more about how veterinarians define a “good” and “bad” euthanasia. The title of the study is Canine and Feline Euthanasia Study.

If you are a veterinarian who euthanizes dogs and/or cats, we would like to ask you to take this anonymous online survey. Participation will take approximately 5-10 minutes. Your participation in this research is voluntary. If you decide to participate in the study, you may withdraw your consent and stop participation at any time without penalty.

We will not collect your name or personal identifiers. When we report and share the data to others, we will combine the data from all participants. While there are no direct benefits to you, we hope to gain more knowledge on how view various aspects of the euthanasia process to build recommendations for best practices for the veterinary industry.

It is not possible to identify all potential risks in research procedures, but the researcher(s) have taken reasonable safeguards to minimize any known and potential (but unknown) risks. If you have any questions about the research, please contact Lori Kogan at lori.kogan@colostate.edu. If you have any questions about your rights as a volunteer in this research, contact the CSU IRB at: RICRO_IRB@mail.colostate.edu; 970-491-1553.This study is funded by the Companion Animal Euthanasia Training Academy (CAETA) and Vortech Pharmaceuticals.
Lori Kogan Professor, Colorado State University

If you consent to complete this survey, please click “Yes I consent” below, to begin the survey.

- ○ Yes, I consent to participating in this research project
- ○ No, I do not want to participate in this research project

---

Are you a veterinarian who performs euthanasia for dogs or cats?

- ○ Yes
- ○ No

---

In what country do you live?

- ○ United States
- ○ Canada
- ○ United Kingdom
- ○ Australia
- ○ Other: ________________________________________________

---

How long have you been practicing veterinary medicine?

- ◯ Less than 5 years
- ◯ 5-10 years
- ◯ Longer than 10 years

---

What year did you graduate from veterinary school?

▼ 1945 ... 2022

---

Approximately how many euthanasias have you facilitated?

- ◯ Less than 500
- ◯ 500-1000
- ◯ Over 1000

---

Please indicate your response to the following statements:

| | Not at all/minimal | Some | Quite a bit | Very/A great deal |
|---|---|---|---|---|
| Your comfort level with the medical aspects of facilitating euthanasia | ○ | ○ | ○ | ○ |
| How well you feel your veterinary training/school prepared you to perform the medical aspects of euthanasia | ○ | ○ | ○ | ○ |
| Your comfort level with the emotional aspects of euthanasia, including communication demands | ○ | ○ | ○ | ○ |
| How well you feel your veterinary training/school prepared you for the emotional and communication aspects of euthanasia | ○ | ○ | ○ | ○ |

When thinking about veterinary curricula, how important do you feel education about euthanasia techniques and euthanasia communication are compared with other clinical skills?

- ◯ Less important
- ◯ Equally important
- ◯ More important

---

Please indicate how frequently you use the following euthanasia techniques to administer euthanasia solution:

| | Never/rarely | Sometimes | Frequently/always |
|---|---|---|---|
| Intravenous | ◯ | ◯ | ◯ |
| Intracardiac | ◯ | ◯ | ◯ |
| Intrahepatic | ◯ | ◯ | ◯ |
| Intrarenal | ◯ | ◯ | ◯ |
| Intraperitoneal | ◯ | ◯ | ◯ |
| Oral | ◯ | ◯ | ◯ |

---

Some techniques used to administer euthanasia solution may be more difficult for clients to witness than others. Please indicate how difficult you feel the following techniques may be for clients to witness:

| | Not at all difficult | Somewhat difficult | Very difficult |
|---|---|---|---|
| Intravenous | ○ | ○ | ○ |
| Intracardiac | ○ | ○ | ○ |
| Intrahepatic | ○ | ○ | ○ |
| Intrarenal | ○ | ○ | ○ |
| Intraperitoneal | ○ | ○ | ○ |
| Oral | ○ | ○ | ○ |

Please share why you feel the intravenous administration method of euthanasia solution is difficult for clients to witness:

________________________________________

Please share why you feel the intracardiac administration method of euthanasia solution is difficult for clients to witness:

________________________________________

Please share why you feel the intrahepatic administration method of euthanasia solution is difficult for clients to witness:

________________________________________________________________________

Please share why you feel the intrarenal administration method of euthanasia solution is difficult for clients to witness:

________________________________________________________________________

Please share why you feel the intraperitoneal administration method of euthanasia solution is difficult for clients to witness:

________________________________________________________________________

Please share why you feel the oral administration method of euthanasia solution is difficult for clients to witness:

________________________________________________________________________

Please rank the following physical symptoms (in isolation with no other symptoms) in order of medical concern when facilitating euthanasia (1 = least concern, 5 = greatest concern):
Click and drag your selection in the order you prefer.

______ Breathing difficulty
______ Low blood pressure
______ Hyperalgesia (extreme sensitivity to pain)
______ Obesity
______ Peripheral edema

Please indicate any other physical symptoms that cause you concern when facilitating euthanasia:

________________________________________________

Please rank the following traits in order of medical concern when facilitating euthanasia (1 = least concern, 5 = greatest concern):

Click and drag your selection in the order you prefer.
_______ Brachycephalic conformation
_______ Short peripheral veins (common in short-legged species)
_______ Excessive skin
_______ Predisposition for disease
_______ Temperament

Please indicate any other traits that cause you concern when facilitating euthanasia:

________________________________________________

Euthanasia is one of the few medical procedures in which clients are permitted to observe. How do you prefer clients be involved with euthanasia?

- ○ Not present at all
- ○ Present only for pre-euthanasia sedation, leave before the euthanasia injection
- ○ Present for everything
- ○ No preference

Please indicate why you prefer clients not be present:

________________________________________________

Please indicate how important you feel the following are in creating a good euthanasia experience:

| | Not at all/minimally important | Somewhat important | Very important |
|---|---|---|---|
| Pre-euthanasia appointments (e.g., discussions around what euthanasia entails, grief support, and general preplanning) | ○ | ○ | ○ |
| Patient pre-visit pharmaceuticals (sedatives given before the appointment) | ○ | ○ | ○ |
| Euthanasia attendants (a consistent person with the client for the duration) | ○ | ○ | ○ |

Please indicate how important you feel the following items are in creating a positive pre-euthanasia appointment:

| | Not at all/minimally important | Somewhat important | Very important |
|---|---|---|---|
| Establish rapport with client and patient | ○ | ○ | ○ |
| Provide materials about grief and loss resources | ○ | ○ | ○ |
| Explain the process of euthanasia | ○ | ○ | ○ |
| Discuss the cost | ○ | ○ | ○ |
| Discuss after-death body care | ○ | ○ | ○ |
| Provide written materials about euthanasia process | ○ | ○ | ○ |

Where do you facilitate euthanasia? (check all that apply)

- ☐ At the veterinary hospital/clinic
- ☐ At clients' homes
- ☐ Other (explain) ____________________________________________________

Below is a list of things that are part of an at-home euthanasia. Please indicate how important you feel the following items are in creating an overall ‘good death’ experience:

| | Not at all important | Slightly important | Moderately important | Very important | Extremely important |
|---|---|---|---|---|---|
| Arriving on time | ○ | ○ | ○ | ○ | ○ |
| Coming prepared with all the materials needed | ○ | ○ | ○ | ○ | ○ |
| Having a professional looking vehicle that can transport a pet | ○ | ○ | ○ | ○ | ○ |
| Being friendly and providing a relaxed atmosphere | ○ | ○ | ○ | ○ | ○ |
| Being willing to gather where the pet wants to be (e.g., on the bed) | ○ | ○ | ○ | ○ | ○ |
| Calling ahead and telling your client what time you are coming | ○ | ○ | ○ | ○ | ○ |
| Respecting your client’s desire to keep the experience private | ○ | ○ | ○ | ○ | ○ |
| Appearing professional | ○ | ○ | ○ | ○ | ○ |
| The pet not afraid upon seeing you | ○ | ○ | ○ | ○ | ○ |
| Helping the client to plan for the presence of other pets | ○ | ○ | ○ | ○ | ○ |
| Providing private time for the client before and after the euthanasia | ○ | ○ | ○ | ○ | ○ |

The following questions pertain to the use of pre-euthanasia sedatives or anesthetics. Sedatives (e.g. butorphanol, midazolam, acepromazine) place pets in a light to deep state of sleep. Anesthetics (e.g. ketamine, propofol, alfaxalone) can induce complete unconsciousness. Herein they will be referred to as pre-euthanasia drugs.

---

How often do you give pre-euthanasia drugs before the euthanasia procedure itself?

- ○ Rarely/never
- ○ Some of the time
- ○ Most of the time
- ○ Always

---

Please indicate how often you use the following methods of administration of pre-euthanasia drugs:

| | Never/rarely | Sometimes | Frequently/always |
|---|---|---|---|
| Orally | ○ | ○ | ○ |
| Injection under the skin and/or muscle | ○ | ○ | ○ |
| Injection via a vein/catheter | ○ | ○ | ○ |

---

Please indicate how relaxed/asleep animals typically are after you give pre-euthanasia drugs.

- ○ Relaxed but awake and responsive
- ○ Relaxed in a light plane of sleep
- ○ Fully unconscious

Typically, how much time is there between when you give the pre-euthanasia drugs and the euthanasia injection?

- ○ Less than 1 minute
- ○ 1-3 minutes
- ○ More than 3 minutes but less than 10 minutes
- ○ 10-20 minutes
- ○ More than 20 minutes

Please indicate how important the following factors are when deciding which pre-euthanasia drugs to use:

| | Not at all/minimally important | Somewhat important | Very important |
|---|---|---|---|
| Patient response to the injection or administration | ○ | ○ | ○ |
| Length of time to effect | ○ | ○ | ○ |
| Added cost | ○ | ○ | ○ |
| Patient health before euthanasia | ○ | ○ | ○ |
| Euthanasia technique options (i.e., intraorgan injections) | ○ | ○ | ○ |

Please indicate how important you feel the following items pertaining to pre-euthanasia drugs are in creating an overall ‘good death’ experience for your clients.

| | Not at all important | Slightly important | Moderately important | Very important | Extremely important |
|---|---|---|---|---|---|
| Explaining the reason for the drugs before given | ○ | ○ | ○ | ○ | ○ |
| Explaining the immediate result of the drugs (how the pet will react) before given | ○ | ○ | ○ | ○ | ○ |
| Allowing the client to be with their pet when the drugs are given | ○ | ○ | ○ | ○ | ○ |
| The drugs' likelihood of medically complicating the procedure | ○ | ○ | ○ | ○ | ○ |
| Necessary restraint to give the drugs | ○ | ○ | ○ | ○ | ○ |
| The drugs taking quick effect | ○ | ○ | ○ | ○ | ○ |

The following is a list of potential effects of a pre-euthanasia drug injection in a patient. Please indicate how much the occurrence of each item would negatively impact your perception of a 'good death':

| | No negative impact | Small negative impact | Moderate negative impact | Large negative impact | Extreme negative impact |
|---|---|---|---|---|---|
| Cause a seizure | | | | | |
| Cause loss of bodily function (urinate, defecate) | | | | | |
| Cause look of confusion | | | | | |
| Cause dizziness or ataxia | | | | | |
| Cause patient to look startled | | | | | |
| Cause look of fear or being scared | | | | | |
| Appear to cause pain | | | | | |
| Cause patient to cry out or vocalize in some manner | | | | | |

The following is a list of potential aspects of euthanasia solution administration. Please indicate how much the occurrence of each item would negatively impact your perception of a ‘good death’.

| | No negative impact | Small negative impact | Moderate negative impact | Large negative impact | Extreme negative impact |
|---|---|---|---|---|---|
| The patient appears scared | ○ | ○ | ○ | ○ | ○ |
| The veterinary team has to restrain the patient | ○ | ○ | ○ | ○ | ○ |
| The patient seems stressed | ○ | ○ | ○ | ○ | ○ |
| The patient’s death appears abrupt | ○ | ○ | ○ | ○ | ○ |
| The patient appears to be in pain in their final moments | ○ | ○ | ○ | ○ | ○ |
| The client does not have an opportunity to be physically close to their pet during last minutes | ○ | ○ | ○ | ○ | ○ |
| The patient’s death is not as fast as you expect | ○ | ○ | ○ | ○ | ○ |
| The veterinary team has to give more euthanasia solution after the first injection or resort to another method | ○ | ○ | ○ | ○ | ○ |
| The veterinary team can not easily find a vein | ○ | ○ | ○ | ○ | ○ |
| The patient is vocal when the euthanasia solution is injected | ○ | ○ | ○ | ○ | ○ |
| The patient thrashes or makes sudden movements when the euthanasia solution is | ○ | ○ | ○ | ○ | ○ |

| | | | | | |
|---|---|---|---|---|---|
| injected | | | | | |
| The veterinary team has to deviate from the plan but the reason is explained to the client | ○ | ○ | ○ | ○ | ○ |

The following is a list of potential reactions patients can have during the euthanasia procedure and death. Please indicate how much the occurrence of each item would negatively impact your perception of a ‘good death’.

| | No negative impact | Small negative impact | Moderate negative impact | Large negative impact | Extreme negative impact |
|---|---|---|---|---|---|
| Seizure | ○ | ○ | ○ | ○ | ○ |
| Urinate/defecate | ○ | ○ | ○ | ○ | ○ |
| Regurgitate/vomit | ○ | ○ | ○ | ○ | ○ |
| Agonal breathing (deep reflexive breaths) | ○ | ○ | ○ | ○ | ○ |
| Body stretching | ○ | ○ | ○ | ○ | ○ |
| Muscle twitches | ○ | ○ | ○ | ○ | ○ |
| Tongue protruding out of the mouth | ○ | ○ | ○ | ○ | ○ |
| Eyes stayed open | ○ | ○ | ○ | ○ | ○ |
| Vocalization | ○ | ○ | ○ | ○ | ○ |

The following is a list of things that can happen immediately after a patient's death. Please indicate how much the occurrence of each item would negatively impact your perception of a 'good death'.

| | No negative impact | Small negative impact | Moderate negative impact | Large negative impact | Extreme negative impact |
|---|---|---|---|---|---|
| The patient's body is not handled with respect after its death | ○ | ○ | ○ | ○ | ○ |
| The client is not offered adequate privacy after their pet's death | ○ | ○ | ○ | ○ | ○ |
| The client is not offered adequate time with their pet after its death | ○ | ○ | ○ | ○ | ○ |

The following is a list of aspects of some euthanasia experiences. Please indicate how much the occurrence of each item would negatively impact your perception of a 'good death'.

| | No negative impact | Small negative impact | Moderate negative impact | Large negative impact | Extreme negative impact |
|---|---|---|---|---|---|
| Client asked to pay at the front desk after the euthanasia | ○ | ○ | ○ | ○ | ○ |
| Client not allowed to spend time with their pet beforehand | ○ | ○ | ○ | ○ | ○ |
| Client not allowed to have other pet(s) present | ○ | ○ | ○ | ○ | ○ |
| Client not allowed to have children present | ○ | ○ | ○ | ○ | ○ |
| The process is not explained appropriately to client's children | ○ | ○ | ○ | ○ | ○ |
| Client not allowed to bring their own music, candles, or other items | ○ | ○ | ○ | ○ | ○ |
| Client feeling that the appointment is too short | ○ | ○ | ○ | ○ | ○ |
| Client not allowed to be with their pet during the entire procedure | ○ | ○ | ○ | ○ | ○ |
| Client hearing their pet, in what sounds like distress, if out of sight | ○ | ○ | ○ | ○ | ○ |
| The pet appearing to be in distress when it is returned to the room | ○ | ○ | ○ | ○ | ○ |

Please indicate how important you feel the following are in increasing the likelihood of ‘good death experience?

| | None at all/minimal | Somewhat | A great deal |
|---|---|---|---|
| Patient pre-visit pharmaceuticals (sedatives given before the appointment) | ○ | ○ | ○ |
| Euthanasia attendants (a consistent person with the client for the duration) | ○ | ○ | ○ |
| Home services | ○ | ○ | ○ |
| Pre-planning with the client | ○ | ○ | ○ |
| Personnel with advanced euthanasia training | ○ | ○ | ○ |
| Veterinary team's communication skills | ○ | ○ | ○ |

Have you had a negative euthanasia experience due to medical/technical difficulties?

○ No

○ Yes (please explain): ________________________________________________

Please indicate any/all ways you have provided support to clients following a negative euthanasia experience:

- [ ] Phone call
- [ ] Email
- [ ] Follow up visit
- [x] No action taken
- [ ] Other ______________________________

---

What action(s) did you take after the negative euthanasia for future euthanasia? Choose all that apply.

- [ ] Altered the technical procedure
- [ ] Switched to different drugs
- [ ] Changed client-present policies
- [x] No action taken
- [ ] Other ______________________________

---

Do you perceive any barriers to making changes to your euthanasia protocols in your veterinary service?

- ○ No
- ○ Yes (please explain): ______________________________

---

Please answer these last questions about you.

How do you identify yourself? (select all that apply)

- ☐ African American/Black
- ☐ Asian
- ☐ Biracial/Multiracial
- ☐ Middle Eastern
- ☐ Native American/Indigenous
- ☐ Native Hawaiian/Pacific Islander
- ☐ White/Caucasian
- ☐ I prefer to self-describe
- ☐ I prefer not to say

What is your ethnicity?

- ○ Hispanic or Latino
- ○ Not Hispanic or Latino
- ○ I prefer to not say

What is your age?

▼ Under 30 years of age ... I prefer to not say

How do you identify?

▼ Female ... I prefer to not say

Please share anything else you want us to know about your euthanasia experiences.

________________________________________________

Thank you for your time; we value your opinion.
